# University Series of School Books

**Published:** 1875-02

**Authors:** 


*University Series of School Books.*

In this number of the Record will be found an advertisement of the University Series of School Books. The names of such eminent educators as Maury, Venable, Holmes, Gildersleeve and others would seem to us sufficient guarantee of the merits of the books; but when we know that they are endorsed by so many of our practical and thorough teachers, who are now using them, and have given them the test of the school-room, we have a double assurance of their positive merits. These books are—all of them—well gotten up, and they are well illustrated and attractive. The people of the South, especially, should feel proud of the enterprise of the Company to build up a literature for them, which is unsectional and unsectarian; and they should feel glad to have the opportunity of encouraging such an enterprise by having the books used in their schools. But the use of the books is not limited to the South, for such works as are so deservedly meritorious, are now used in many of the best schools throughout the Union, and Maury and Gildersleeve are authority across the Atlantic.

The purpose of the University Publishing Company is so commendable and important, that it has received the cordial endorsement of *thousands* of the best men of the country—among whom are Gen. J. E. Johnston, Gen. John B. Gordon, Bishop George F. Pierce, of Georgia; Bishop R. H. Wilmer, Hon. Thomas H. Watts, Alabama; Bishop Marvin, Missouri; Hon. John C. Brown, (Governor) Tennessee; Rev. John A. Waddell, D.D., (Chancellor University) Mississippi; Gen. Z. B. Vance, North-Carolina, etc., etc.

We indorse the enterprise of the Company and their books, and shall do what we can to put them into our schools. We have heretofore been dependent on other sections for school-book literature, but the necessity no longer exists, and we hope to be able to state, at another time (for we shall have more to say of the books, etc.) that our people are at last appreciating the efforts of the University Publishing Company to furnish them with such excellent school books by Southern authors.

